# Profiling the expression and functional roles of mRNAs and lncRNAs associated with post-stroke aphasia

**DOI:** 10.3389/fnmol.2025.1513218

**Published:** 2025-04-09

**Authors:** Yanling Xi, Hui Chang, Mei Qu

**Affiliations:** ^1^Department of Rehabilitation Medicine, Shanghai Pudong New Area Guangming Hospital of Traditional Chinese Medicine, Shanghai, China; ^2^School of Foreign Languages, Shanghai Jiao Tong University, Shanghai, China

**Keywords:** post-stroke aphasia, long non-coding RNA, messenger RNA, RNA sequencing, expression profiles

## Abstract

**Objective:**

Post-stroke aphasia (PSA) is one of the primary causes of post-stroke impairment, although its underlying mechanism is unknown; therefore, this study aimed to identify the long non-coding RNAs (lncRNAs) and messenger RNAs (mRNAs) linked to PSA and to understand the potential processes by which they may operate.

**Methods:**

RNA sequencing was used to determine the lncRNA and mRNA expression profiles for PSA patients and healthy control peripheral blood mononuclear cells. This allowed for the discovery of lncRNAs and differentially expressed genes (DElncRNAs and DEGs). Gene Ontology (GO) and KEGG enrichment analyses were performed on these DElncRNAs and DEGs, and qPCR was used to confirm their expression. Furthermore, any correlations between these characteristics with differential expression and the language routines of PSA patients were evaluated.

**Results:**

In total, comparisons of the groups yielded 577 DElncRNAs and 892 DEGs. Functional enrichment analyses of these targets demonstrated the strong enrichment of co-expressed DElncRNAs and DEGs in immune system processes and the inflammatory response. The expression levels of the lncRNAs CTD-2545M3.2 and RP11-24N18.1 and the mRNAs RPS10 and LAIR2 were similarly highly connected with verbal conduct in PSA patients upon admission.

**Conclusion:**

The results highlight the lncRNA and mRNA profiles linked to PSA, demonstrating the various methods via which these DElncRNAs and DEGs may influence this clinical setting.

## Introduction

Stroke is a leading global driver of death and disability throughout the globe (Yu et al., 2023). Post-stroke aphasia (PSA) is a prevalent and significant consequence of stroke, impacting approximately 30% of patients in the acute phase and 10–18% in the chronic phase (Jang et al., 2022). PSA adversely affects patients’ linguistic skills and ability to communicate (Zhang et al., 2021). Therefore, finding PSA-related biomarkers may offer a more effective way to identify the condition early on and aid in developing more effective diagnostic and treatment approaches.

High-throughput sequencing methodologies have facilitated the identification of numerous hematological biomarkers at the transcriptome level, aiding in identifying diseases and patient prognosis evaluation (Bilchick et al., 2020; Fernández Pérez et al., 2022). Peripheral blood mononuclear cells (PBMCs) are immune cells that include lymphocytes and monocytes (Chen et al., 2020). These PBMCs are valuable candidates for transcriptome analysis while monitoring PSA patients because of the unique changes in blood biochemistry associated with stroke-induced brain injury (Aldous et al., 2022). These alterations can affect gene expression patterns in PBMCs and are mostly linked to inflammation and neuroplasticity following a stroke. However, plasma is a biofluid that is easier to obtain than tissue samples. These factors make PBMCs a potentially useful source of PSA-related indicators.

To date, no research has have systematically evaluated the messenger RNA (mRNA) and long non-coding RNA (lncRNA) expression profiles associated with PSA or defined the underlying mechanisms through which they function. This study aimed to clarify the expression patterns and functional functions by comparing PBMCs of PSA patients with those of healthy controls, thereby enhancing the understanding of PSA pathophysiology to inform future prevention and therapy strategies.

## Materials and methods

### Assessment of language behavior

A solitary professional language therapist employed the Chinese Aphasia Battery (CAB) to assess the linguistic capabilities of all participating patients. The standardized ABC is the most often used instrument for evaluating aphasia in China. It is a dependable and valid version of the Western Aphasia Battery (WAB) for Chinese culture. Participants in this test were assessed on their oral expression, listening comprehension, reading, writing, application, structure, visual spatial skills, and numeration abilities. Behavioral evaluations for patients with PSA were conducted during admission and discharge.

### PBMC isolation

Peripheral blood samples were obtained upon hospital admission from 8 (3 males and 5 females) healthy controls (HCs), 9 (5 males and 4 females) stroke patients, and 12 patients (7 males and 5 females) with PSA, with post-treatment samples (PSA-T) collected from all 12 PSA patients at discharge. PBMCs were isolated from the blood samples using Ficoll density gradient centrifugation and stored at −80°C for future analysis.

This study received approval from the Shanghai Pudong New Area Guangming Hospital of Traditional Chinese Medicine ethics committee and was in line with the Declaration of Helsinki. Written informed consent was obtained from all participants in this study.

### RNA extraction

TRIzol (15596018, Invitrogen, USA) was used according to the manufacturer’s instructions for RNA extraction from PBMCs. This process included two rounds of phenol-chloroform treatment for RNA purification, followed by DNA removal using RQ1 DNase (M6101, Promega, WI, USA). Absorbance at 260/280 nm (A260/A280) was measured using an Ultrafine spectrophotometer (N50 touch, IMPLEN, Germany) to determine the quantity and quality of the extracted RNA. At the same time, its integrity was assessed via 1.0% agarose gel electrophoresis.

### RNA sequencing (RNA-seq) library preparation

Wuhan Ruixing Biotechnology Co., Ltd. conducted RNA-seq analyses. In brief, 1 μg of RNA from all samples underwent treatment with RQ1 DNase (M6101, Promega, USA) to eliminate DNA before the preparation of a directional RNA-seq library using the VAHTS® Universal V8 RNA-seq Library Prep Kit for Illumina (NR605, Vazyme, China). The capture of mRNA was accomplished using VAHTS mRNA capture beads (N401, Vazyme, China). RNAs that had been fragmented were then converted into double-stranded cDNA, and end repair and A tailing were performed. These DNAs were then ligated to the VAHTS RNA Multiplex Oligos Set 1 for Illumina (N323, Vazyme, China), amplified, purified, quantified, and stored at −80°C pending sequencing analysis. The second cDNA strand labeled with dUTP was amplified to enable strand-specific sequencing analyses. High-throughput sequencing was conducted according to the manufacturer’s instructions, employing 150 nt paired-end sequencing on an Illumina Novaseq 6000 instrument.

### Differential expression analyses

DESeq2 was employed to model raw reads, using scale factors to account for variations in library depth. Gene dispersion was subsequently assessed using DESeq2, enhancing the precision of dispersion estimates for read count modeling via shrinkage application. DESeq2 was also used to fit a negative binomial distribution model, while likelihood ratio or Wald tests were employed for hypothesis testing. Differential expression analyses were then performed to identify differentially expressed genes (DEGs) and differentially expressed long non-coding RNA (DElncRNAs) between PSA and HC samples based on the following: fold change (FC) ≥1.5 or ≤0.6667 and *p* < 0.05.

### Predicted mRNA and lncRNA co-expression interactions

Co-expression analyses for DElncRNAs and DEGs were conducted by evaluating Pearson correlation coefficients between lncRNAs and genes pairs in the analyzed samples. LncRNA-mRNA pairs with a *r* > 0.6 and a *p*-value <0.01 were retained, as they were deemed to demonstrate co-expression interactions. Assessing cis-regulatory relationships involved setting a co-localization threshold range for regulatory pairs within 100 kb upstream or downstream of a specific lncRNA. Subsequent co-expression analyses used Pearson correlation coefficients, selecting significant lncRNA-mRNA pairs (*r* > 0.6, *p* ≤ 0.01). The overlap between the co-localized and co-expressed datasets was then used to establish cis-regulatory lncRNA targets.

### Functional enrichment analyses

Target genes associated with DElncRNAs were identified through cis and trans analyses. Cis target genes were protein-coding genes within 100 kb upstream or downstream of a given lncRNA. Coding gene expression levels were used to determine trans-target genes. Trans-targets were classified as genes with substantial Pearson correlation coefficients (*r* > 0.6, *p* < 0.01) with the lncRNA. The roles of co-expressed DElncRNAs and DEGs were investigated using Gene Ontology (GO) and KEGG analyses. LncRNA targets were mapped to GO and KEGG terms using DAVID. A hypergeometric distribution test was conducted using the GO and KEGG annotations for the entire genome as the background, and the numbers of genes linked to each term/pathway were ascertained to identify the GO and KEGG words that were significantly enriched. GO terms with FDR < 0.05 were enriched considerably after the *p*-values were adjusted using the Benjamini-Hochberg method. Following that, the top 10 enriched terms and pathways for these lncRNA targets were selected for further presentation.

### qPCR

RNA-seq results were validated by randomly selecting 6 DEGs and 10 DElncRNAs from the PSA versus HC comparison and assessing their expression via qPCR. The 3 DEGs and 3 DElncRNAs demonstrating the most significant expression differences between groups were selected for qPCR examination of their expression in the PSA-T and stroke groups and the PSA and HC groups. TRIzol (15596018, Invitrogen) was used as directed to prepare RNA from PBMCs, after which a reverse transcription kit (R323-01, Vazyme, China) was used to prepare cDNA with a Mycycler instrument (T100, Bio-Rad, USA) as follows: 42°C for 5 min, 37°C for 15 min, 85°C for 5 s. Then, qPCR analyses were performed with an ABI QuantStudio 5 instrument as follows: 95°C for 10 min; 40 cycles of 95°C for 15 s, and 60°C for 1 min. Samples were prepared with three technical replicates, GAPDH served as a normalization control, and results were analyzed via the 2^−ΔΔCT^ method. Primer sequences (3′–5′) were as follows:

- SNHG25-F: CGTCGGATGTCATCGTCCTT,
- SNHG25-R: CGCAAGCACATGCTCTAGC;
- RP1-56 K13.3-F: TCCAAACATTTCATCCGTGTGT,
- RP1-56 K13.3-R: AGAAAGGTTTGATCCCGAGCA;
- RP11-339H12.2-F: GTCCAAAGTAGTCCAGAGAG,
- RP11-339H12.2-R: ATGTAGAAAGGCAGGAATGT;
- CTD-2545 M3.2-F: CCACCCTCCCTGTAAGTCTAT,
- CTD-2545 M3.2-R: CGTATCAAGTGAGCAGTGAATT;
- RP11-69H7.3-F: GAGAACACACATGGGCTTTGG,
- RP11-69H7.3-R: GGCTCAGCAGAGGTGAAGTT;
- RP11-326 K13.5-F: ACTGGGAAGAACTGAGGTTTC,
- RP11-326 K13.5-R: TGATGGCAAACTAAGGTTCAGA;
- RP11-24 N18.1-F: TTGCCCTCCCATCCTCTCA,
- RP11-24 N18.1-R: AAGTCCTTGTCCAGCCATCC;
- RP11-104 L21.2-F: CCTGTGTTCGACTCATCTTACG,
- RP11-104 L21.2-R: TTGCTGAAAGTAGCGCAGTTT;
- RP11-473C18.7-F: TGATGGCAAGGTGTGGTCTAC,
- RP11-473C18.7-R: CGAAACCCACCCGAGAACA;
- RP11-732A19.10-F: CATTTAGGTTGGGTGGCTCAG,
- RP11-732A19.10-R: ACCGCAAGTACACTCTAACCA;
- LCN2-F: GGAGAACCAAGGAGCTGACTT,
- LCN2-R: GATTGGGACAGGGAAGACGAT;
- LAIR2-F: AACATTCCGCCTGGAGAGG,
- LAIR2-R: TCACTGTGCTCAGACCATCC;
- ATG10-F: GAGAAACATTGGCTACTTTGAC,
- ATG10-R: AATGATGAAGTTCTGGTTGCTG;
- RPS10-F: GACATGAAGGTTGGGCACATT,
- RPS10-R: GCCTAAGAAGAACCGGATTGC;
- ALAS2-F: GCACACAACAAAGCAGAAGAC,
- ALAS2-R: CCAGTATGTCACCACCTATGC;
- HBB-F: AGTTGGACTTAGGGAACAAAGG,
- HBB-R: GTGGCTGGTGTGGCTAATG;
- GAPDH-F: GGTCGGAGTCAACGGATTTG,
- GAPDH-R: GGAAGATGGTGATGGGATTTC.

### Statistical analyses

Correlations between the lncRNA and mRNA expression were examined by correlation analysis. Data were analyzed using GraphPad Prism (v9.1.2, GraphPad Software, CA, USA) and are shown as means ± standard deviation.

## Results

### Identification of differentially expressed PSA-associated genes and lncRNAs

**Figure 1:**
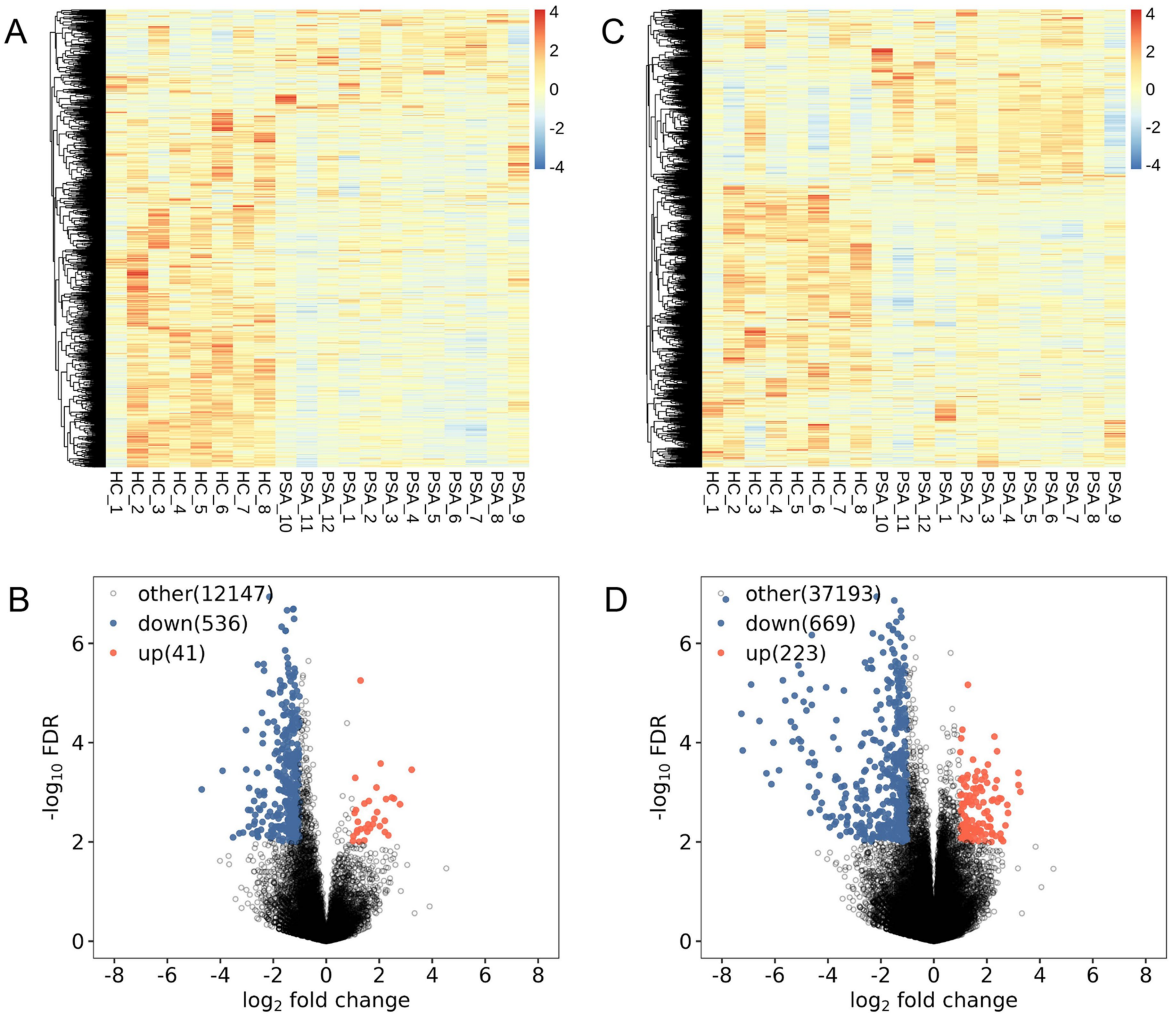
Differential expression analyses. **(A,C)** Differential lncRNA **(A)** and mRNA **(C)** clustering heatmaps, with red and blue, respectively, denoting transcripts expressed at high and low levels. **(B,D)** Volcano plots show differentially expressed lncRNAs **(B)** and mRNAs **(D)**, with red and blue denoting significantly upregulated and downregulated transcripts.

Volcano plots and heatmaps were used to depict DElncRNAs (Figures 1A,B) and DEGs (Figures 1C,D). Overall, 577 DElncRNAs (41 were upregulated) and 893 DEGs (223 were upregulated) were found in PSA patients’ PBMCs.

### Functional enrichment analyses of lncRNA targets for co-expressed DElncRNAs and DEGs based on lncRNA trans-regulation

**Figure 2:**
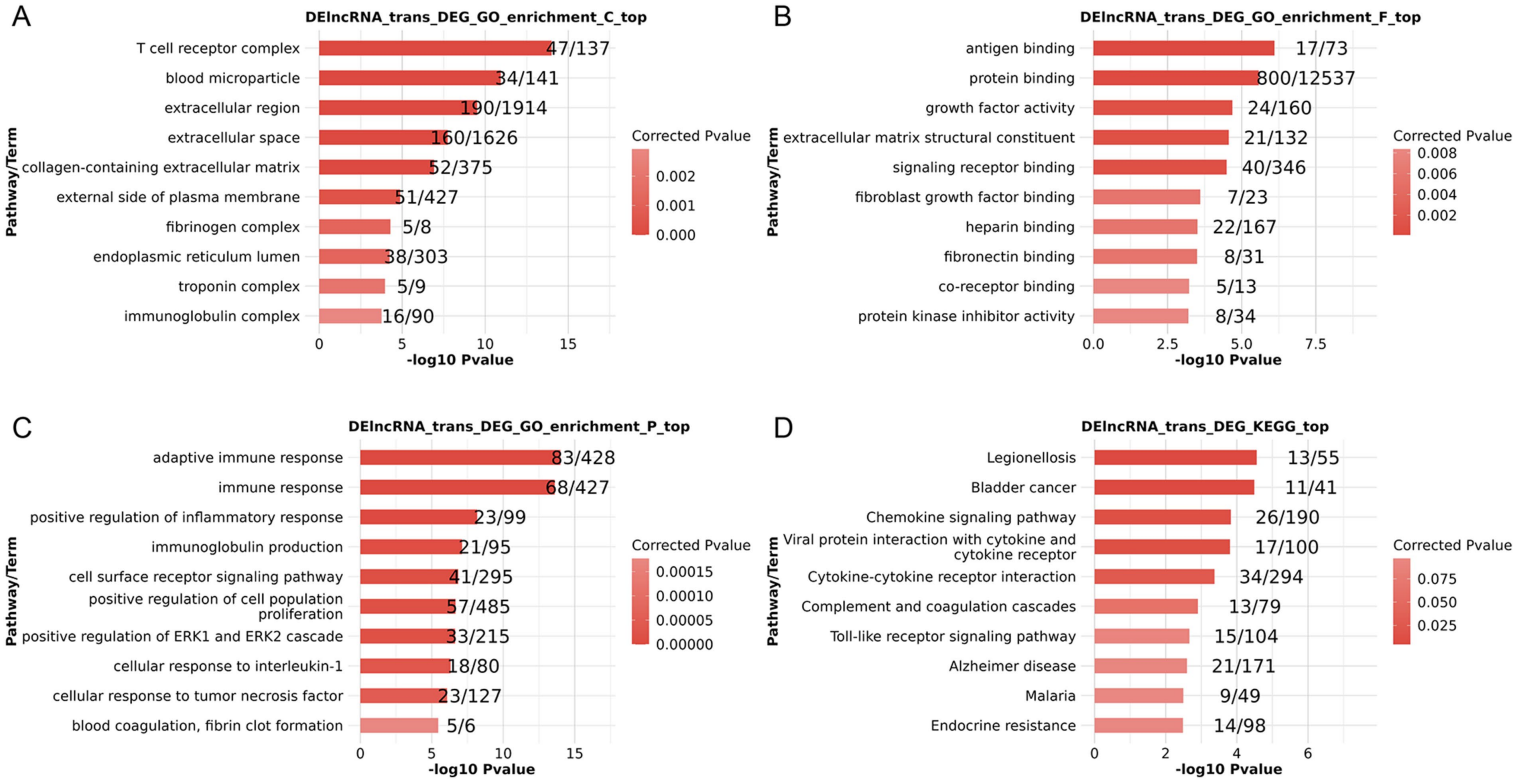
Functional enrichment analyses of lncRNA targets for co-expressed DElncRNAs and DEGs based on lncRNA trans-regulation. **(A–C)** GO CC **(A)**, MF **(B)**, and BP **(C)** enrichment results. **(D)** KEGG pathway enrichment results.

When GO enrichment analyses were conducted on co-expressed DElncRNAs and DEGs, the results showed that several enriched cellular component (CC), molecular function (MF), and biological process (BP) terms were found. T cell receptor complex, blood microparticles, and the extracellular matrix were among the enriched CC terms (Figure 2A). Enriched MF terms included antigen binding, protein binding, and growth factor activity (Figure 2B). Enriched BP terms included the adaptive immune response, positive regulation of the inflammatory response, and cellular responses to IL-1 and TNF (Figure 2C). Enriched KEGG pathways included chemokine signaling, Toll-like receptor signaling, and Alzheimer’s disease (Figure 2D). Therefore, via regulating immunological function, extracellular matrix connections, and disease signaling pathways, these co-expressed DElncRNAs and DEGs may affect the onset of PSA.

### Functional enrichment analyses of lncRNA targets for co-expressed DElncRNAs and DEGs based on lncRNA cis-regulation

**Figure 3:**
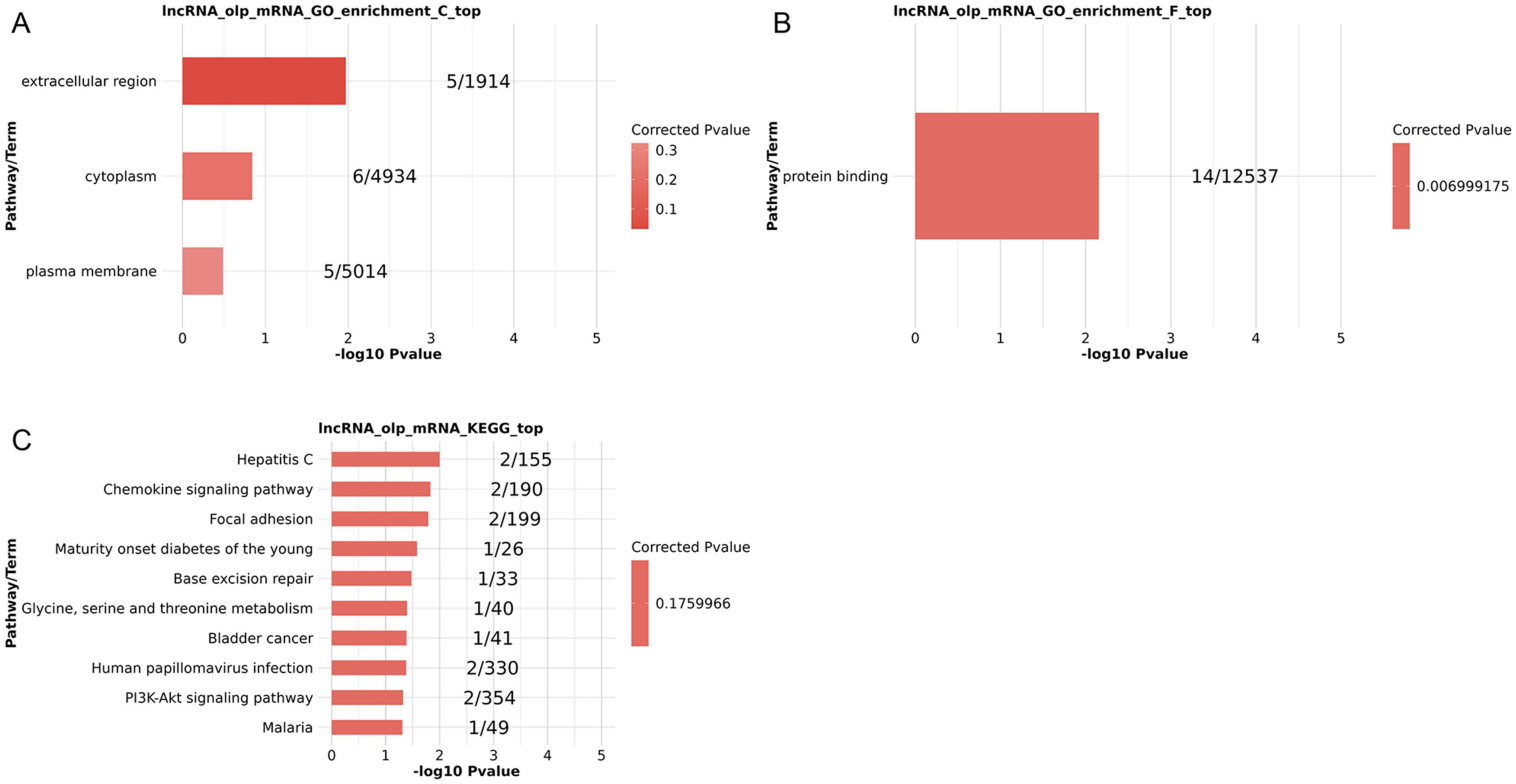
Functional enrichment analyses of lncRNA targets for co-expressed DElncRNAs and DEGs based on lncRNA cis-regulation. **(A–C)** GO CC **(A)** and MF **(B)** enrichment results. **(C)** KEGG pathway enrichment results.

GO and KEGG enrichment analysis of co-expressed DElncRNAs and DEGs revealed their correlation with several biological terms when focusing on the cis-regulatory targets of DElncRNAs. Protein binding was an enriched MF term (Figure 3B), while the extracellular region, cytoplasm, and plasma membrane were enriched CCs (Figure 3A). Hepatitis C, chemokine signaling, focal adhesion, maturity-onset diabetes of the young, base excision repair, glycine, serine and threonine metabolism, bladder cancer, human papillomavirus infection, Pl3K-Akt signaling, and malaria pathways were among the other KEGG pathways that were discovered to be enriched (Figure 3C). These results suggest that cis-regulated genes and co-expressed lncRNAs may be strongly linked to the pathophysiology of PSA by influencing immune-related processes, potentially through PI3K-Akt and/or chemokine signaling pathways and/or protein binding.

### Identified DElncRNA and DEG validation

**Figure 4:**
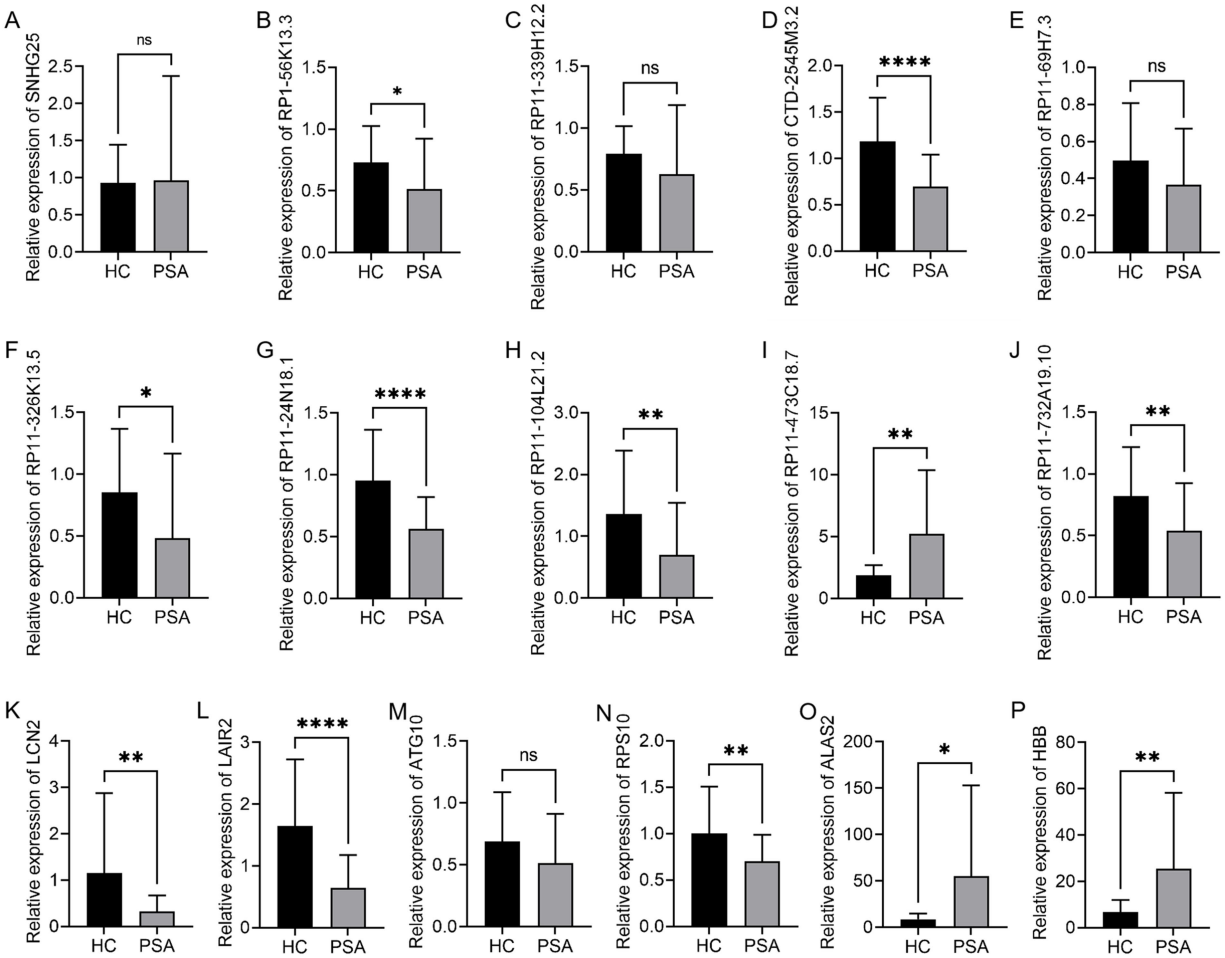
qPCR-based validation of DEGs and DElncRNAs. qPCR was used to confirm relative levels of expression for **(A)** SNHG25, **(B)** RP1-56K13.3, **(C)** RP11-339H12.2, **(D)** CTD-2545M3.2, **(E)** RP11-69H7.3, **(F)** RP11-326K13.5, **(G)** RP11-24N18.1, **(H)** RP11-104L21.2, **(I)** RP11-473C18.7, **(J)** RP11-732A19.10, **(K)** LCN2, **(L)** LAIR2, **(M)** ATG10, **(N)** RPS10, **(O)** ALAS2, and **(P)** HBB, with GAPDH as a normalization control (*p < 0.05, **p < 0.01, ***p < 0.001, ****p < 0.0001).

The accuracy of these bioinformatics analyses was confirmed by randomly selecting 10 DElncRNAs and six DEGs. Their expression levels in HC and PSA samples were measured using a qPCR method. The 10 DElncRNAs included eight that were downregulated (SNHG25, RP1-56K13.3, RP11-339H12.2, CTD-2545M3.2, RP11-69H7.3, RP11-326K13.5, RP11-24N18.1, RP11-104L21.2) and two that were upregulated (RP11-473C18.7, RP11-732A19.10). For the six DEGs, five were downregulated (LCN2, LAIR2, ATG10, RPS10, ALAS2), and one was upregulated (HBB). These targets other than RP11-732A19.10, SNHG25, RP11-339H12.2, RP11-69H7.3, and ATG10 exhibited good consistency with the expected expression patterns. Specifically, RP1-56K13.3, CTD-2545M3.2, RP11-326K13.5, RP11-24N18.1, RP11-104L21.2, and RP11-732A19.10 were significantly downregulated in PSA samples, whereas RP11-473C18.7 was significantly upregulated (Figures 4A–J). Of the analyzed DEGs, LCN2, LAIR2, RPS10, and ALAS2 were significantly downregulated in PSA samples, while HBB was significantly upregulated (Figures 4K–P). These findings further support these factors as mediators of PSA development.

**Figure 5:**
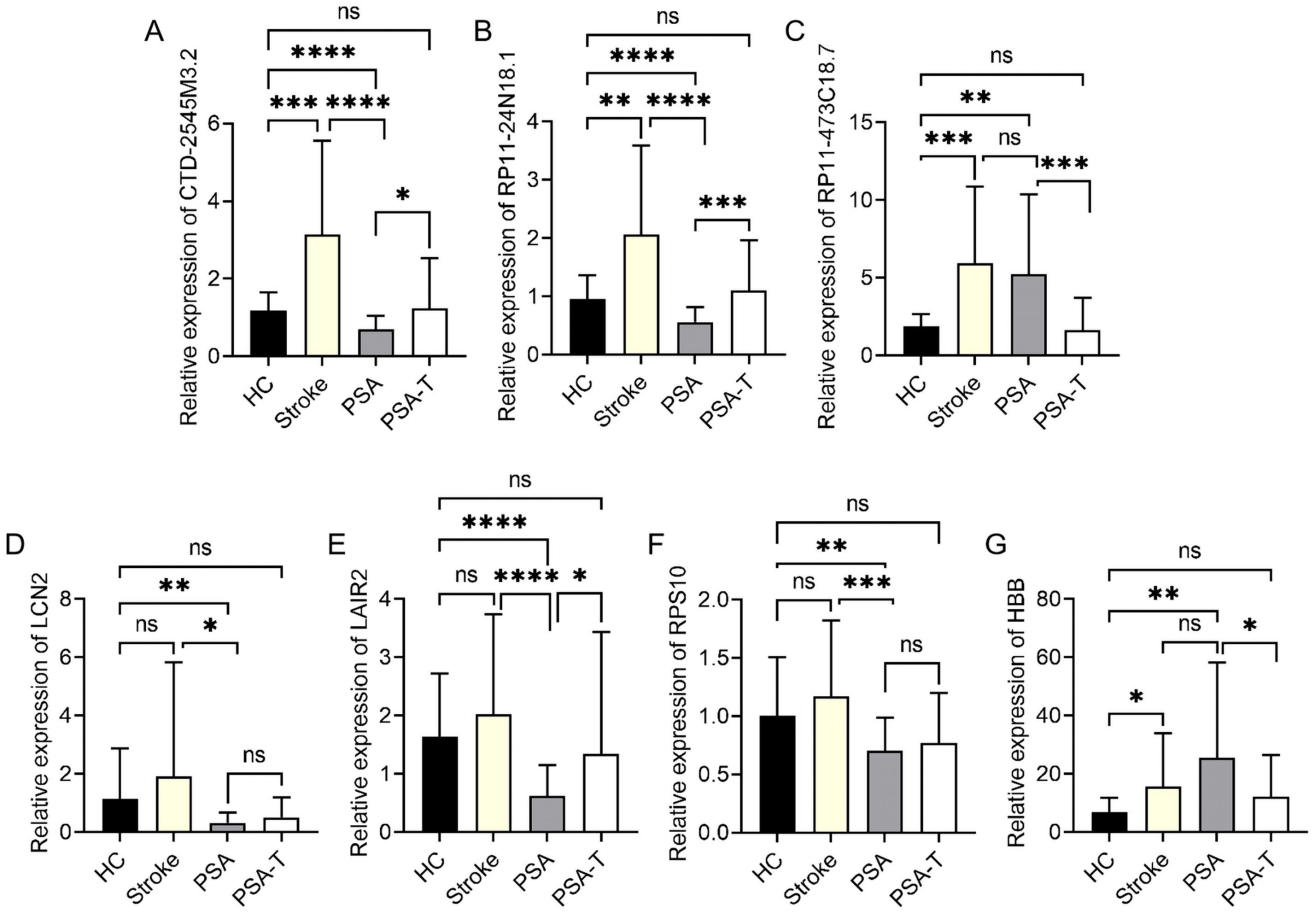
qPCR-based analysis of target mRNA and lncRNA expression in specific patient groups. **(A–G)** qPCR was employed to assess relative expression levels of **(A)** CTD-2545M3.2, **(B)** RP11-24N18.1, **(C)** RP11-473C18.7, **(D)** LCN2, **(E)** LAIR2, **(F)** RPS10, and **(G)** HBB, with GAPDH as a normalization control (*p < 0.05, **p < 0.01, ***p < 0.001, ****p < 0.0001).

The identified DElncRNAs and DEGs were further validated to understand better how these molecules changed as the stroke progressed, PSA developed, and treatment was administered. Furthermore, their potential as biomarkers for disease monitoring and therapy evaluation was investigated. In particular, the three DElncRNAs (CTD-2545M3.2, RP11-24N18.1, and RP11-473C18.7) and four DEGs (LCN2, LAIR2, RPS10, and HBB) that showed the most significant variations between PSA and HC samples were selected for further qPCR verification in the samples from the HC, stroke, PSA, and PSA-T groups. In this analysis, CTD-2545M3.2, RP11-24N18.1, and RP11-473C18.7 were significantly upregulated in stroke samples relative to HC samples. At the same time, CTD-2545M3.2 and RP11-24N18.1 but not RP11-473C18.7 were significantly downregulated in the PSA group relative to the stroke group. All three lncRNAs differed when comparing the PSA and PSA-T groups (Figures 5A–C). When comparing samples from the HC and stroke groups, the expression of only one of the examined mRNAs, HBB, varied. At the same time, HBB levels stayed high, although PSA samples from stroke patients showed significantly lower levels of LCN2, LAIR2, and RPS10 (Figures 5D–G). No significant differences in these DEGs or DElncRNAs were observed when comparing the HC and PSA-T groups.

### Examination of the correlative relationships between DElncRNAs, DEGs, and language behavior

**Figure 6:**
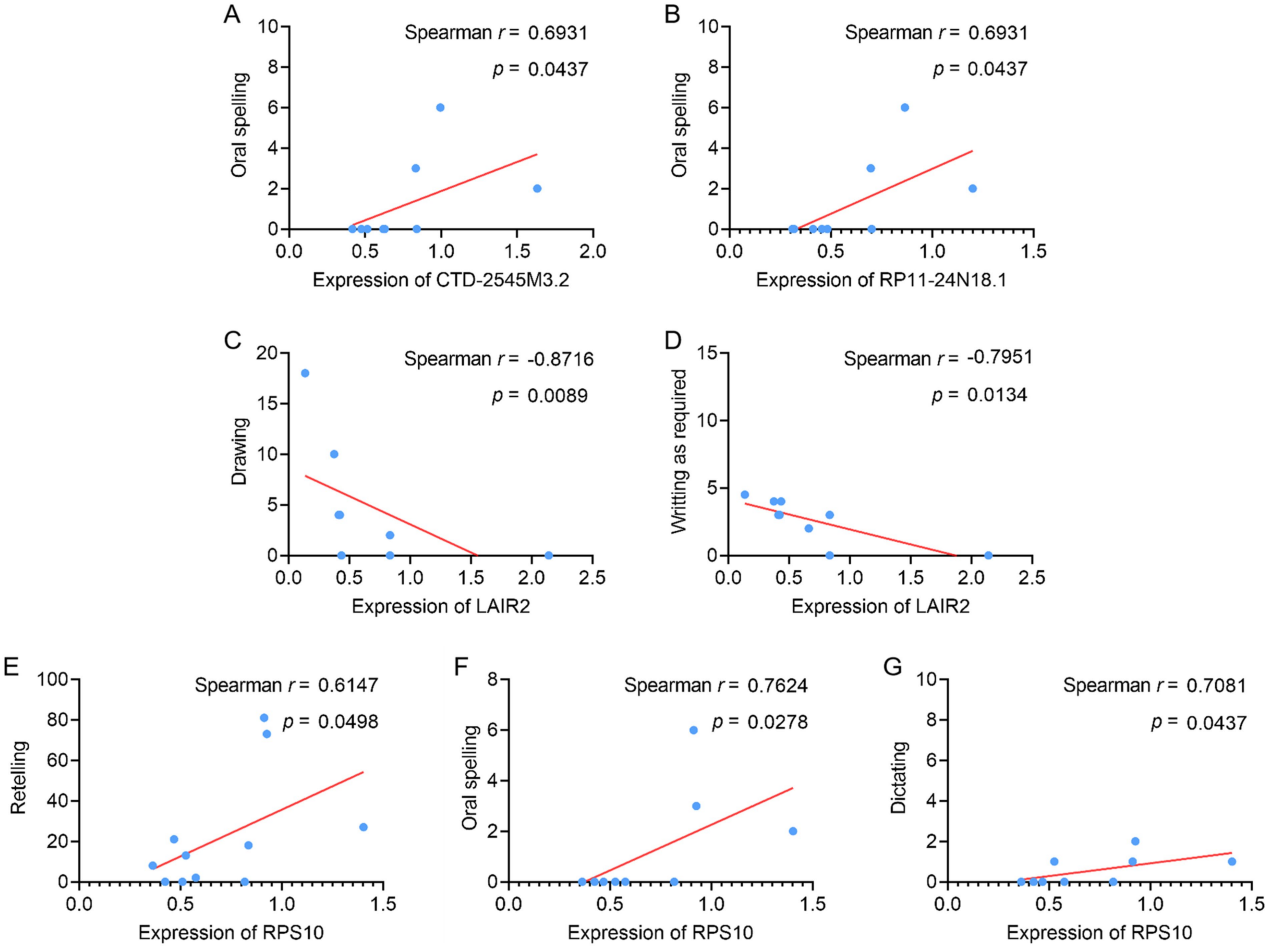
Assessment of correlations between PSA patient language behavior test scores and specific DElncRNAs and DEGs. **(A–G)** Correlations were assessed between **(A)** oral spelling and CTD-2545M3.2 levels, **(B)** oral spelling and RP11-24N18.1 levels, **(C)** drawing and LAIR2 levels, **(D)** writing as required and LAIR2 levels, **(E)** retelling and RPS10 levels, **(F)** oral spelling and RPS10 levels, and **(G)** dictating and RPS10 levels.

The relationship between the expression levels of related molecules and the language behavior test scores of PSA patients was examined to determine whether there is a correlation between the differentially expressed lncRNAs and mRNAs and the language function of these patients, as well as the direction and strength of this association. Correlation analyses revealed that CTD-2545M3.2 and RP11-24N18.1 levels in patients with PSA were positively correlated with oral spelling scores (Figures 6A,B), whereas LAIR2 levels were negatively correlated with writing upon request and drawing scores (Figures 6C,D). However, compared to HC, PSA patients had lower levels of LAIR2 in both RNA-seq and qPCR analyses. Furthermore, RPS10 positively correlated with dictation, oral spelling, and repetition scores (Figures 6E–G). These results offer novel perspectives on the pathophysiology of PAS and raise the possibility that these molecules could be used as biomarkers for early identification and evaluation.

## Discussion

PSA is a condition in which cerebrovascular disease leads to acquired cognitive language impairment (Wang et al., 2020), presenting in the form of disrupted oral fluency, naming, repetition, comprehension, reading, and/or writing (Flowers et al., 2016). Although numerous methods exist for treating aphasia, speech and language therapy is the most often used (Wang et al., 2020). The current bottleneck in PSA treatment relates to uncertainty about treatment efficacy and the variability among individual cases. Therefore, more efficacious therapeutic techniques are required. The present study evaluated alterations in PBMC lncRNA and mRNA expression profiles using an RNA-seq technique in HC and PSA samples, identifying 577 DElncRNAs and 892 DEGs.

Co-expression analyses of DEGs and DElncRNAs, based on the regulator effects of DElncRNAs in trans, identified multiple PSA-related enriched GO BP terms associated with immunity, inflammatory response, and neural repair processes. Stroke pathogenesis is strongly connected to neuroinflammation and immunosuppression (Jiang et al., 2021). Adaptive immunity and antibody production can contribute to more severe neuroinflammation, culminating in neurological dysfunction (Hu et al., 2023), thus affecting language recovery. Neural repair also highly depends on ERK signaling pathway (Cotto-Rios et al., 2020; Singh et al., 2020). Inflammatory mediators are also closely involved in post-stroke pathology (McCabe et al., 2021). However, co-expression analyses of lncRNA cis-regulatory relationships did not identify enriched BP terms. This could be because certain DEGs and lncRNAs have weaker associations or lncRNAs functions are unclear, making it impossible to identify any significance associated with specific processes of interest. Therefore, it is important to properly investigate these lcnRNAs’ possible impacts on PSA since they may participate in cis-regulatory actions that are different from the mechanisms via which they operate in trans.

Key biological pathways linked to PSA were highlighted by KEGG pathway enrichment analysis conducted for both trans- and cis-regulatory interactions. These inflammatory and immunological reactions were discovered to be crucial at the beginning of PSA in terms of trans-regulatory interactions. Microglia-mediated neuroinflammation is a characteristic of neurodegenerative disorders such as Parkinson’s and Alzheimer’s (Song and Suk, 2017). Numerous pro-inflammatory chemokines and cytokines secreted by M1 microglia can cause inflammation and neuronal death (Guo et al., 2022). Inflammatory cytokine infiltration is also closely tied to the pathogenesis of neurological diseases linked to impaired sensorimotor functions and cognition (Eve et al., 2022). Signaling through Toll-like receptor-4 can also mediate autoimmunity and neuroinflammatory activity in the context of neurodegenerative diseases (Zhang et al., 2020). The PI3K-Akt signaling, base excision repair, human papillomavirus infection, and maturity-onset diabetes of the young were among the enriched pathways concerning cis-regulatory linkages. Diabetes may increase the risk of PSA because it has been previously associated with increased rates of stroke and a higher risk of more severe neurological disorders (Grisotto et al., 2021; Huang et al., 2019). Effectively repairing damaged DNA and protecting against cellular death may also positively affect diseases linked to central nervous system damage (Luo et al., 2022). PI3K-Akt signaling activity is closely tied to cellular survival and neuroplasticity (Matsuda et al., 2019; Saw et al., 2020; Xin et al., 2017). Activating this pathway can initiate brain healing that enhances recovery from PSA. Collectively, these mechanistic insights enhance understanding of the intricate pathological foundation of PSA, guiding the identification of suitable targets for therapeutic intervention.

LncRNAs serve as essential regulators of many different biological processes, and they have also been linked to brain disease onset in humans (Yu et al., 2023). The upregulation of RP11-473C18.7 and the downregulation of CTD-2545M3.2 and RP11-24N18.1 were confirmed, while qPCR-based validation efforts failed to detect the expression of all analyzed lncRNAs accurately. Both CTD-2545M3.2 and RP11-24N18.1 showed a significant positive correlation with verbal spelling scores of PSA patients, suggesting that they may be involved in PSA onset and thus be used as a new biomarker. The precise functions of most lncRNAs are still unknown because functional annotations are lacking.

LCN2 encodes a protein in the lipocalin superfamily, also known as neutrophil gelatinase-associated lipocalin (NGAL), which is linked to ferroptotic cell death. There is evidence linking LCN2 to the exacerbation of ischemic stroke in an STZ-induced diabetes model system, with such toxicity being attributable to neutrophil-derived ferroptosis. Since lncRNAs can function through a variety of post-transcriptional mechanisms, including roles as miRNA regulators or effectors, they can easily shape target mRNA expression in the context of PSA (Wang et al., 2024). LCN2 levels were significantly lower in PSA patients than in HCs, indicating that it may control the incidence of PSA. It has also been demonstrated that the ribosomal protein RPS10 is linked to inflammatory reactions and neurodegeneration (Zhong et al., 2024). The observed RPS10 downregulation in participants with PSA was positively related to reading ability, demonstrating the functional relevance of this protein in the context of PSA. LAIR2 is a soluble leukocyte-associated immunoglobulin-like receptor associated with variations in the activity of T cells (Pan et al., 2023). However, HBB is related to immune cell infiltration (Zhang et al., 2023). The specific pathways connecting LCN2, RPS10, LAIR2, and HBB to PSA incidence and progression have yet to be explored.

In contrast to the RNA-seq and qPCR results, correlation analysis showed a negative correlation between LAIR2 and PSA patient language behavior ratings. Individual differences between samples could cause this variability, leading to uneven gene expression levels and functions. Increased statistical variability, may also result from smaller sample sizes, explaining discrepancies in the relationship between behavioral traits and gene expression levels.

Several factors could limit the interpretation of these results. Firstly, the sample size was restricted, necessitating future large-scale validation. Future research should incorporate a more extensive cohort of PSA patients, reflecting a variety of demographic factors, including aphasia severity levels. Secondly, prospective cohort studies could be undertaken to validate the identified biomarkers further and their potential roles in PSA pathogenesis and recovery. Thirdly, although there was no significant difference in gender composition among the groups, this study primarily focused on the differential expression profiles of lncRNAs and mRNAs between groups. The potential influence of gender on expression profiles was not further explored, which may have masked possible gender-specific expression differences. Future studies should include gender as an independent variable to determine whether key lncRNAs and mRNAs exhibit gender-specific expression differences. Moreover, differential expression profiles of PSA-related mRNA and lncRNA were found via RNA sequencing and bioinformatics methods, with the identified targets predominantly associated with inflammatory, immunological, and other functional pathways. Direct molecular tests will be essential to confirm the applicability of these findings to the clinical context. Translating these observations into animal models is challenging due to considerable neuroanatomical differences, mainly the lack of language-specific regions such as Broca’s and Wernicke’s areas in rodents. Future research should prioritize functional experiments and explore alternative models to address the complexity of PSA in human brain regions more effectively. Furthermore, it is essential to recognize the limitations associated with using bulk RNA-seq from PBMCs. PBMCs consist of various immune cell types, and the gene expression changes observed may indicate the cumulative effects of multiple cell populations. Future research could be acquired by isolating distinct immune cell types, such as monocytes or T cells, to elucidate the cellular mechanisms associated with PSA accurately. Advanced neuroimaging, single-cell RNA sequencing, and spatial transcriptomics could enhance understanding of these biomarkers’ molecular dynamics and spatiotemporal regulation in post-stroke recovery.

## Conclusion

In conclusion, this study identifies a variety of PSA-related DEGs and DElncRNAs that could be connected to the development and course of the illness via immunological and inflammatory processes. In particular, the RPS10 and LAIR2 genes, as well as the lncRNAs CTD-2545M3.2 and RP11-24N18.1, were found to be important potential regulators of PSA. These findings provide a solid basis for developing novel therapeutic targets and diagnostic indicators for PSA management.

## Data Availability

The raw data supporting the conclusions of this article will be made available by the authors without undue reservation.
